# Changeover method for biosafety cabinets using ozone gas

**DOI:** 10.1371/journal.pone.0318006

**Published:** 2025-01-28

**Authors:** Mitsuru Mizuno, Daisuke Suda, Chima Matsumura, Ichiro Sekiya

**Affiliations:** 1 Center for Stem Cell and Regenerative Medicine, Institute of Science Tokyo, Bunkyo-ku, Tokyo, Japan; 2 Biozone Medical Co., Ltd., Fujisawa-shi, Kanagawa, Japan; University of Jeddah, SAUDI ARABIA

## Abstract

This study evaluated the effectiveness of a biosafety cabinet equipped with an ozone generator, particularly during the transition periods between the production of cell products. As living cell products cannot undergo sterilization, maintaining an aseptic manufacturing environment is paramount. Raw materials, often derived from human tissues, are frequently contaminated with various resident bacteria, necessitating environmental resets after each process. The utility of this device against bacteria, including endotoxins, endospores, and fungi endemic to human tissues, could facilitate safe and reproducible production changeovers through a simplified, one-button operation. This study focused on bacteria resistant to conventional cleaning protocols, specifically targeting endospore-forming bacteria with robust resistance to disinfectants, spore-forming fungi, and included analyses of endotoxins. The effects of ozone exposure on *Pseudomonas aeruginosa* (an endotoxin-producing bacterium), *Bacillus subtilis* (an endospore-forming bacterium), and *Aspergillus brasiliensis* (a spore-forming fungus) were assessed. In the dedicated biosafety cabinet equipped with an ozone generator, the treatment group exposed to ozone showed a significant reduction in both colony-forming units and endotoxin levels in *Pseudomonas aeruginosa* at 1.0 × 10^4^ colony-forming units (CFUs) compared to the control group. Moreover, the ozone treatment markedly decreased the colony formation of *Bacillus subtilis* endospores and *Aspergillus brasiliensis* spores. Given its effectiveness against endospores and endotoxins—among the most challenging bacterial derivatives to eliminate—the device demonstrates potential for enhanced bacterial control in Grade A biosafety cabinets within cell product manufacturing facilities. The system may substantially reduce operator stress by ensuring product safety through straightforward operational procedures and high reproducibility, although further validation is required.

## Introduction

Maintaining environmental sterility is essential for the safe and effective processing of living cell products. Consequently, protecting these products from risks such as contamination by environmental bacteria within cell processing facilities is crucial [1–4]. Additionally, it is essential to prevent contamination from residual materials such as culture medium droplets [5–8]. Consequently, the choice of changeover method after each operation becomes critical in safeguarding cell products. Conventional changeover techniques include wiping with disinfectant and fogging with hydrogen peroxide. However, manual disinfection by operators introduces concerns regarding reproducibility and reliability. These concerns are particularly pronounced in manufacturing environments that must comply with stringent good manufacturing practice (GMP) standards. The lack of objective criteria for assessing wiping effectiveness makes it difficult to ensure consistent reliability [6]. Additionally, operators often question the thoroughness of the cleaning and disinfection processes, which can lead to mental stress [9]. The effectiveness of disinfectants against highly resistant bacteria, such as endospore-forming bacteria, depends on the grade of the disinfectant, with stronger disinfectants being more effective but potentially more corrosive and odorous, posing challenges for operators [10]. Additionally, materials derived from human sources frequently carry various pathogens [11,12], and the complete elimination of unknown pathogens is not feasible. Therefore, manual wiping also poses a risk of pathogen exposure for operators. In this context, fogging with hydrogen peroxide offers a promising alternative, as it can be implemented by simply setting up the equipment, thereby minimizing operator exposure to pathogens. However, the presence of residual substances in the biosafety cabinet after fogging poses significant concerns regarding cell viability. For instance, studies have demonstrated that residual hydrogen peroxide can still affect the proliferation and survival of human bone marrow-derived mesenchymal stem cells up to 30 h post-fogging [13]. While UV irradiation is straightforward and highly reproducible, it fails to reach shaded areas effectively. Consequently, the development of an optimal changeover method that balances the advantages and disadvantages of existing techniques remains an unresolved issue.

This study aimed to evaluate the utility of a biosafety cabinet fitted with an ozone generator for changeovers in the production of cell products. Recent research has highlighted the effectiveness of ozone gas in neutralizing bacteria and fungi [14–16]. Ozone possesses strong oxidizing capabilities and attacks the cell walls and membranes, thereby disintegrating proteins and nucleic acids [17,18]. Unlike conventional antibacterial agents that target specific metabolic pathways, ozone acts through a mechanism that is less prone to induce resistance [19]. Its instability and rapid decomposition also facilitate its use across various applications. However, the potential adverse effects on human health, such as loss of consciousness at elevated concentrations, present substantial challenges to its application [20,21]. This issue has particularly complicated its use in open workspaces, including biosafety cabinets. Despite these concerns, biosafety cabinets with fully automated ozone generators are increasingly utilized for changeovers involving anticancer drugs, thereby enhancing operator safety [22]. Designed to reduce operator exposure to hazardous substances, this device incorporates a one-button operation system to streamline the process. Our research aimed to confirm the effectiveness of this system under conditions that mimic real-world changeovers during cell product manufacturing.

This study focused on bacteria and bacterial-derived substances that pose significant challenges for eradication through standard cleaning methods. Our focus included endospore-forming bacteria known for their high resistance to disinfection and spore-forming fungi. Additionally, we targeted endotoxins, notorious for their difficulty in disinfecting during changeover procedures. We chose *Bacillus subtilis (B. subtilis)* as the representative endospore-forming bacterium, identified in the pharmacopoeias of various countries as a potential contaminant in biosafety cabinets and a crucial target for ensuring the sterility of cell products [23–27]. Endotoxins, consisting of lipopolysaccharides that make up the cell walls of Gram-negative bacilli, are released as pyrogens upon the death or mechanical disruption of the bacteria. Pharmaceutical guidelines establish standard thresholds for endotoxins in the shipping of cell products, pharmaceuticals, and medical devices [28,29]. These Gram-negative bacilli, containing endotoxins, can inadvertently be introduced into a biosafety cabinet along with human tissue-derived raw materials. For instance, cell product materials such as epidermal and intestinal epithelial cells may enter a Grade A environment viable and persist within the biosafety cabinet. In this context, we selected *P. aeruginosa*, recognized in pharmacopoeia as an indicator bacterium, to study the effects of ozone exposure on its endotoxin production and viability. Similarly, we evaluated the activity of *Aspergillus brasiliensis (A. brasiliensis)*, a pharmacopoeial indicator fungus known for its spore resistance to various disinfectants. We assessed its ability to form colonies after ozone exposure, measuring colony-forming unit (CFU) formation.

## Materials and methods

### Experimental conditions

**Fig 1:**
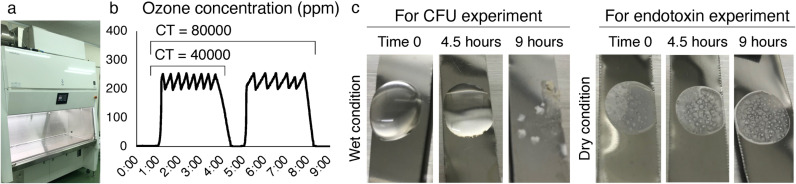
Experimental setup. (A) Medio3 biosafety cabinet equipped with an ozone generator. (B) Ozone generation trends over time, where the x-axis represents time (h), and the y-axis represents ozone concentration in parts per million (ppm). CT denotes concentration-time, calculated as the product of ozone concentration and exposure duration. (C) Experiments for colony-forming units (CFU) and endotoxins conducted under wet and dry conditions, respectively.

In this study, we utilized a biosafety cabinet equipped with an ozone generator (Medio3; BIOZONE MEDICAL Co., Ltd., Toyama, Japan) as depicted in Fig 1A. Ozone concentration trends were continuously monitored (Fig 1B). The experimental ozone exposure was regulated based on the concentration-time (CT) value, defined to ensure the decomposition of anticancer drugs overnight [22]. The 8 h requirement is substantiated by various procedural factors. Initially, 1 h is necessary for preparatory measures, which include elevating the humidity to 80% to optimize ozone efficacy and conducting a leak test to verify that the cabinet maintains proper pressure. Continuous exposure to 250 ppm of ozone, and an additional hour is allotted for the decomposition of residual ozone. While the device is capable of higher ozone concentrations, the selected concentration optimally balances operational effectiveness and maintainability.

CFU experiments were conducted under wet conditions, whereas endotoxin assessments were carried out under dry conditions. Bacterial solutions were prepared in 50 µL of physiological saline (Otsuka Pharmaceutical Co. Ltd., Tokushima, Japan) and placed on stainless steel tape; in the wet conditions, ozone exposure commenced immediately, whereas, in the dry conditions, 10 µL of physiological saline with bacteria was left stationary overnight prior to exposure (Fig 1C). Control groups were situated in a biosafety cabinet in the same room under identical conditions and for the same durations as the treatment groups.

### CFU measurements

To evaluate the impact of ozone gas on bacterial viability, CFUs of two bacterial strains were evaluated. *Pseudomonas aeruginosa (P. aeruginosa)* (ATCC 9027; Microbiologics, Inc., St. Cloud, MN, USA) and *A. brasiliensis* (ATCC 16404; Microbiologics, Inc.) were grown in precultures, diluted to a concentration of 5 × 10^4^ CFU, and plated on stainless-steel surfaces. Additionally, *B. subtilis* endospores (1 × 10^8^ CFU; NBRC 13722; Bioball Multishot 10E8; bioMérieux, Marcy l’Etoile, France) were rehydrated from their lyophilized state to active endospores and similarly plated. Both the experimental and control groups were placed on stainless steel in conical tubes (NIPPON Genetics Co., Ltd., Tokyo, Japan), dispersed in 10 mL of sterile saline, and agitated with a vortex mixer to elute the bacteria and fungi. The eluate was then diluted tenfold in sterile saline, and 100 μL of this dilution was inoculated onto dual plates of soybean casein digest agar (Shimadzu Diagnostics, Inc., Tokyo, Japan) and incubated at 32.5 ± 2.5 °C for up to five days. The number of viable microorganisms was quantified based on colony counts and the dilution factor.

### Endotoxin measurements

Treatment and control groups of *P. aeruginosa* (1 × 10^7^ CFU) were prepared, with the treatment groups subjected to drying and subsequent ozone exposure at CT values of 40000 and 80000. Samples were transferred to stainless-steel plates within conical tubes containing 1 mL of sterile saline and vortexed to elute the bacteria.

Endotoxin concentrations were quantified using the Limulus ES-II single test as per the manufacturer’s instructions (Fujifilm Wako Pure Chemical Industries, Osaka, Japan), employing a toxinometer (ET-6000; Fujifilm Wako Pure Chemical Industries).

### Statistical analyses

Statistical analyses were conducted using Prism version 9 (GraphPad Inc., La Jolla, CA, USA) and R software (R Foundation for Statistical Computing, Vienna, Austria). Data are presented as medians with interquartile ranges (IQRs). Specifics of each statistical test are detailed in the corresponding figure legends, with statistical significance established at p <  0.05.

## Results

### Ozone-exposure effects on *P. aeruginosa*

**Fig 2:**
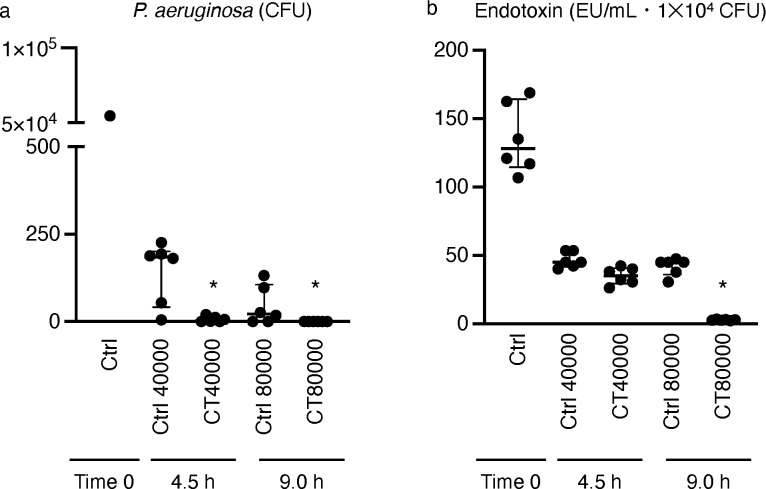
Effect of ozone gas on *P. aeruginosa.* (A) CFU values for control and treatment groups (N = 6), and (B) Endotoxin levels for each group (N = 6). “Ctrl” indicates controlgroup samples maintained in a biosafety cabinet without ozone exposure for durations equivalent to the treatment groups. All data are presented as median values with interquartile ranges (IQRs) and * P < 0.05. P-values were derived using the Kruskal–Wallis test with Steel–Dwass multiple comparison test.

The treatment group of *P. aeruginosa* exposed to ozone gas at CT 40000 demonstrated significantly reduced colony formation (median = 3.5; IQR = 0.0–14.0) compared to the control group (median = 184.5; IQR = 41.8–201.3), which was maintained in a biosafety cabinet for 4.5 h without ozone exposure (Fig 2A). In a similar trend, no colony formation was observed in the treatment group exposed to ozone gas at CT 80000, and colony counts were significantly lower than those in the control group (median = 22.0; IQR = 0.0–105.8), which was kept in a biosafety cabinet for 9 h (Fig 2A).

Regarding endotoxin levels, the treatment group exposed to ozone gas at CT 40000 displayed significantly reduced endotoxin concentrations per 1.0 × 10^4^ CFU (median = 35.2; IQR = 29.5–40.7) compared to the control group maintained in a biosafety cabinet for 4.5 h without ozone exposure (median =  44.9; IQR = 41.8–53.5) (Fig 2B). Similarly, the amount of endotoxins per 1.0 ×  10^4^ CFU in the treatment group exposed to ozone gas at CT 80000 was markedly lower (median = 2.8; IQR = 2.5–3.2) compared to that in the control group, which was kept for 9 h without ozone exposure (median = 44.9; IQR = 36.1–45.6) (Fig 2B).

### Ozone-exposure effects on *B. subtilis* formed with endospores

**Fig 3:**
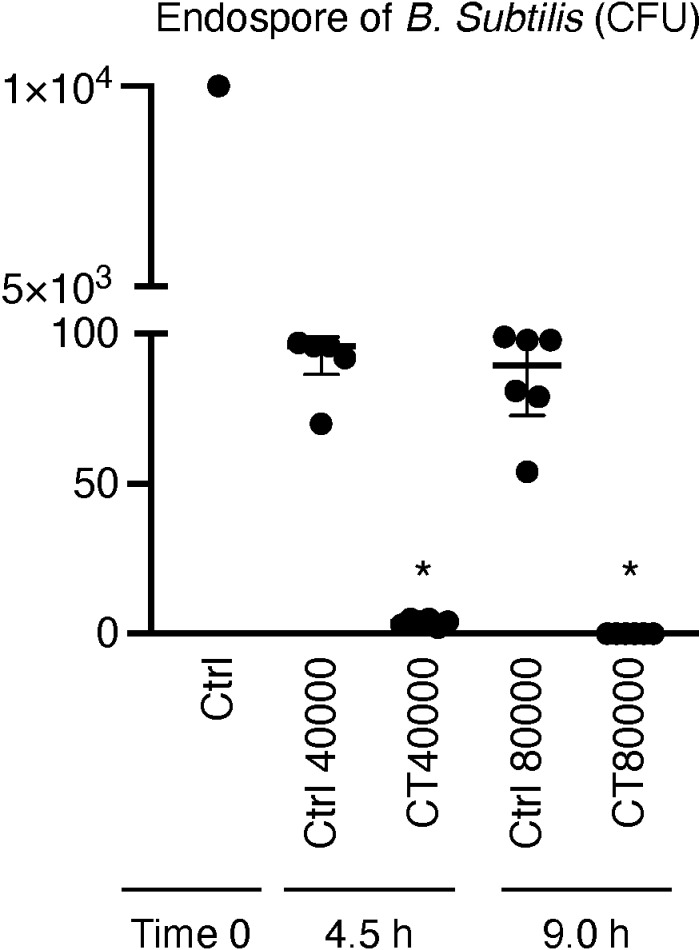
Effect of ozone gas on endospores of *Bacillus subtilis.* CFU values for control and treatment groups (N = 6). Data are shown as median values with IQRs and * P < 0.05. P-values were obtained using the Kruskal–Wallis test with Steel–Dwass multiple comparison test. “Ctrl” denotes controlgroup samples maintained in a biosafety cabinet without ozone exposure for equivalent durations as the treatment groups.

In the case of *B. subtilis*, the treatment group exposed to ozone gas at CT 40000 also showed significantly reduced colony formation (median = 4.0; IQR = 2.8–5.0) relative to the control group (median = 96.0; IQR = 86.5–99.0), which was housed in a biosafety cabinet for 4.5 h without ozone exposure (Fig 3). Furthermore, no colony formation was observed in the treatment group exposed to ozone gas at CT 80000, displaying significantly lower colony counts than the control group (median = 89.5; IQR = 72.8–98.3), which was kept in a biosafety cabinet for 9 h without ozone exposure (Fig 3).

### Ozone-exposure effects on *A. brasiliensis*

**Fig 4:**
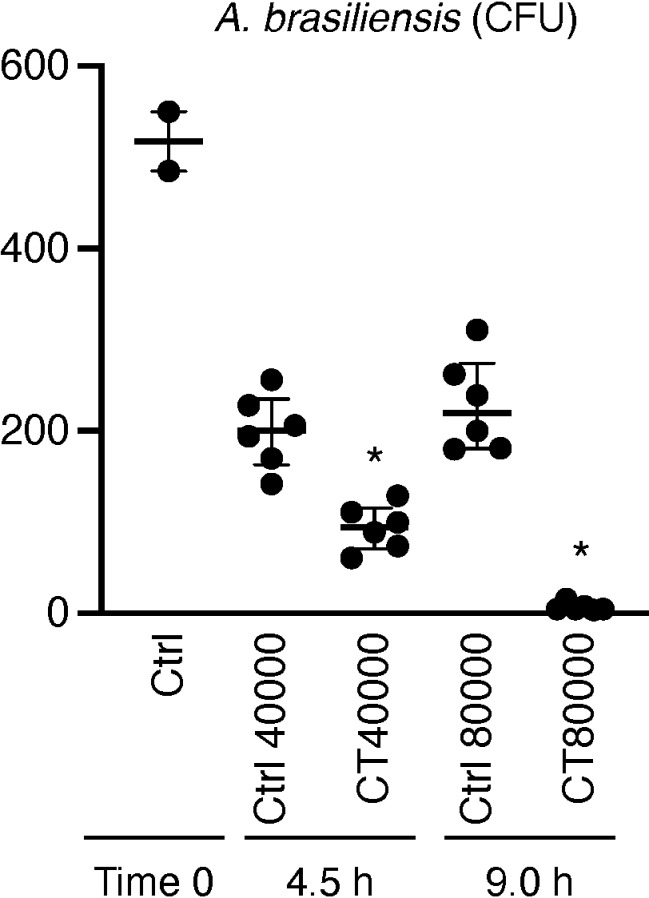
Effect of ozone gas on spores of *A. brasiliense.* CFU values for control and treatment groups (N = 6). Data are presented as median values with IQRs and * P < 0.05. P-values were calculated using the Kruskal–Wallis test with Steel–Dwass multiple comparison test. “Ctrl” refers to controlgroup samples that were kept in a biosafety cabinet without ozone exposure for the same duration as the treatment groups.

The treatment group of *A. brasiliensis* exposed to ozone gas at CT 40000 demonstrated significantly reduced colony formation (median = 94.5; IQR = 70.8–115.5) compared to the control group (median = 200.0; IQR = 163.0–235.0), which was maintained in a biosafety cabinet for 4.5 h without ozone exposure (Fig 4). Additionally, the colony formation was significantly lower in the treatment group exposed to ozone gas at CT 80000 (median = 5.0; IQR = 4.8–10.0) than in the control group (median = 219.5; IQR = 180.8–274.3), which was kept in a biosafety cabinet for 9 h without exposure (Fig 4).

## Discussion

### Summary

Cell products may harbor bacteria, fungi, and substances like endotoxins due to the non-sterilizable nature of the raw materials. Consequently, product-lot changeovers in Grade-A environments become extremely critical processes. These changeovers must ensure the reliability required in cell product production; however, addressing the issue of low reproducibility with a simple method remains unestablished. In this study, we explored the use of ozone gas, recognized for its bactericidal properties that microorganisms find difficult to resist. We examined its effects on residual bacterial and fungal species. A biosafety cabinet equipped with an ozone gas generator and devices that meet GMP reliability standards by automatically documenting ozone exposure was employed. The findings suggest that changeover methods utilizing ozone gas could be seamlessly integrated into cell production facilities following thorough validation.

### Effects of ozone on bacteria and endotoxins

*P. aeruginosa* is a Gram-negative bacterium, while endotoxin, a lipopolysaccharide that forms the cell wall of Gram-negative rods, is a pyrogenic substance released upon the death or mechanical disruption of bacteria. For pharmaceuticals and medical devices, it is crucial to ensure minimal levels of these substances to prevent infections during shipping inspections [28,29]. The genus *Pseudomonas* is rarely detected in clean room environments [30]; however, it is specified as a target bacterium in the pharmacopoeias of various countries, serving as an effective indicator for assessing endotoxin residues. Given that raw materials for cell products, such as intestinal epithelium, are introduced into Grade-A environments in a viable state, they pose a risk of contamination. If intestinal epithelium were employed as the raw material in this study, the experimental setup could represent valid accelerated testing relevant to actual manufacturing conditions. Although techniques such as ultrafiltration and adsorption are effective for removing endotoxins from liquids [31], they are unsuitable for cleaning large surfaces like those of biosafety cabinets. The heating method, which involves carbonizing substances at 250 °C for 30 min or more, is typically used for stainless steel surfaces in biosafety cabinets. However, the extensive work surface of these cabinets, measuring 120 cm × 60 cm, renders this method impractical.

Washing remains the most traditional and commonly used method for decontaminating large objects such as biosafety cabinets [32], although the effectiveness of this approach is variable. For instance, one study indicated that washing surfaces with 70% ethanol could remove endotoxins [33], while other findings suggest that this method does not eliminate endotoxins or prevent inflammatory cytokine production in cultured cells [34]. These inconsistencies highlight the low reproducibility of the cleaning methods. Consequently, identifying practical technologies to remove endotoxins that ensure high reproducibility and reliability remains a challenge, particularly in the production of cell products in compliance with GMP standards. Recently, the application of ozone gas in removing endotoxins has been explored and found effective, especially in maintaining water hygiene—a critical public health issue [35,36]. Although ozone is a potent oxidizer of common chemicals, its high oxidizing potential poses significant risks to human health, making its use in open environments inadvisable. This study utilized a mechanical method to achieve a consistent exposure level, mitigating variations attributable to different techniques and operators. We found that exposure to ozone at a CT value of 80,000 effectively reduced the CFUs in the treatment groups to approximately 1/100th of those in the control groups, which contained 10,000 CFUs—substantially higher than the standard control value of 1 CFU typical for Grade-A environments. These findings indicate that endotoxins produced by various bacteria in these settings can be effectively eliminated.

### Effects of ozone on endospores

*Bacillus* species are prevalent in cell processing facilities [7,30]; therefore, *B. subtilis* serves as a useful indicator organism, targeted in the pharmacopoeias of several countries. *B. subtilis* forms endospores that are highly resistant to disinfection, making it an appropriate species for evaluating methods to eradicate endospores from contaminated biosafety cabinets [6,7]. While wiping and UV irradiation can effectively remove bacteria, the efficacy of wiping may vary and depend on the technique of the individual operators [6]. Although UV irradiation can swiftly eliminate many endospores [7], some operators prefer to maintain air-conditioned environments in biosafety cabinets without UV exposure. In this study, we propose an initialization method for biosafety cabinets that is independent of operational techniques and effectively utilizes non-operating periods. The ozone gas exposure method outlined here can be conducted daily after the use of the biosafety cabinet, thus allowing operations to proceed without the risk of contamination from spore-forming bacteria introduced by raw materials or operators. Moreover, as cleaning is a known stress factor for operators [9], using this method can significantly enhance the working environment, ensuring operator comfort and safety.

### Effects of ozone on fungus

In this study, *A. brasiliensis* [37], a fungus known for its high resistance to disinfection, was selected for validation due to its ability to form spores that, once established, are difficult to eradicate. Notably, once *A. brasiliensis* adheres to a high-efficiency particulate air (HEPA) filter, it becomes entrenched within the filter matrix. The skin epithelium and oral mucosa, used in the cultivation of epidermis and cornea in medical applications, comprise various fungal flora, including *Aspergillus* species [38,39], which raises the potential for contamination of raw materials. If such fungi from raw materials adhere to the work surfaces of biosafety cabinets, their prompt removal becomes imperative. Surface disinfection with ozone gas is recognized for its effectiveness against fungal contaminants [40,41], and in this study, ozone gas exposure was significantly effective in inhibiting fungal colony formation, corroborating findings from previous research. This method is anticipated to be both effective and practical for eliminating the concentrations of bacteria and fungi typically found in cell-product manufacturing environments.

### Limitations

This study presents two principal limitations. First, our analysis was restricted to only three categories—bacteria, fungi, and endotoxins—which may not sufficiently cover the spectrum of potential bacterial contaminants. Although our focus was on endospores, known for their high resistance to disinfectants, it is probable that the methods employed would also be effective against less resistant bacteria. However, further studies are required to investigate the effectiveness of this approach against other bacteria specified in the Pharmacopoeia, such as *Clostridium sporogenes*, *Staphylococcus aureus*, and *Candida albicans*. Additionally, cell product raw materials may contain non-enveloped viruses such as Parvovirus B19, which are resistant to disinfectants [42]. While ethanol often proves ineffective against these viruses, ozone is broadly acknowledged as effective against them [17,18,43]; thus, future validation using ozone gas is warranted. Subsequent research will aim to further refine the adaptability of this device for cell product manufacturing environments.

Second, the necessary equipment incurs significant costs. The systems within this device are fully automated and cannot operate until the ozone has been adequately neutralized through catalytic decomposition. For example, the biosafety cabinet remains locked until ozone concentrations are reduced to safe levels. These systems are critical for ensuring operator safety and maintaining a non-toxic environment suitable for cell culture. However, the requirement to adhere to multiple safety standards significantly elevates the equipment’s cost. Unlike typical biosafety cabinets, this device incorporates advanced safety features, including components designed to resist oxidation and prevent ozone leakage. Moreover, the maintenance and inspection costs of these sensors and other safety components are considerable. Given the current scale of cell therapy production and the manual processes still prevalent in larger facilities, a thorough assessment of cost-benefit ratios is essential.

## Conclusion

We explored a method for initiating changeovers in biosafety cabinets through exposure to ozone gas. The proposed equipment has proven effective against endospores and endotoxins, which are among the most difficult bacterial-derived substances to eliminate. This device facilitates the safe and reproducible initialization of biosafety cabinets with a single-button operation, enhancing safety in cell product manufacturing environments. As the demand for cell product manufacturing is anticipated to increase, the utility of this device is expected to become even more significant, offering a scalable solution for ensuring sterility in critical settings.
